# CircHomer1 may contribute to postoperative cognitive impairment by modulating Homer1b/mGluR5-associated signaling in the vCA1 region of aged mice

**DOI:** 10.3389/fnmol.2026.1859800

**Published:** 2026-07-06

**Authors:** Wei Ran, Yiwei Shen, Ruixue Yuan, Jin Gao, Ning Liang

**Affiliations:** Department of Anesthesiology, The First Affiliated Hospital of Chongqing Medical University, Chongqing, China

**Keywords:** aging, circHomer1, cognitive function, Homer1b, POD-like postoperative neurocognitive impairment

## Abstract

**Background:**

Postoperative delirium (POD) is a common and severe complication in elderly patients after surgery, primarily characterized by a decline in cognitive function.

**Methods:**

Presently, the pathogenesis of POD is still unclear, thus making it imperative to investigate the key mechanisms of its transcriptional regulation. Circular RNAs have been implicated in mental and cognitive disorders. Our previous circRNA profiling suggested that circRNAs may be involved in postoperative cognitive changes. In this study, we used Western blotting, immunofluorescence staining, transmission electron microscopy, and behavioral tests to investigate whether circHomer1 contributes to POD-like early postoperative neurocognitive impairment. In this study, we used Western-blot, immunofluorescence staining, transmission electron microscopy, and animal behavior tests to explore the mechanism by which circRNA mediates the progression of POD.

**Results:**

Three main findings are reported. First, tibial fracture surgery induced significant cognitive impairment in 12–14 month-old mice, as evidenced by reduced freezing time in contextual fear conditioning test and reduced center zone exploration time in open field test on postoperative day 3. Second, siRNA-mediated knockdown of circHomer1 in the ventral CA1 (vCA1) region significantly alleviated surgery-induced cognitive deficits, increased Homer1b mRNA expression, and upregulated mGluR5 and PSD-95 protein levels. Conversely, siRNA-mediated knockdown of Homer1b exacerbated cognitive impairment and reduced mGluR5 and PSD-95 expression, while lentivirus-mediated overexpression of Homer1b rescued both protein levels and cognitive function. Third, pharmacological activation of mGluR5 with the agonist CHPG improved cognitive performance and increased PSD-95 expression, whereas inhibition of mGluR5 with MPEP produced opposite effects.

**Conclusions:**

This study suggests that circHomer1 in the vCA1 region may influence the cognitive function of elderly mice following tibial fracture surgery, potentially involving the Homer1b/mGluR5 signaling pathway, offering preliminary mechanistic insights into the role of circRNAs in brain function and disease.

## Graphical Abstract

**Figure:**
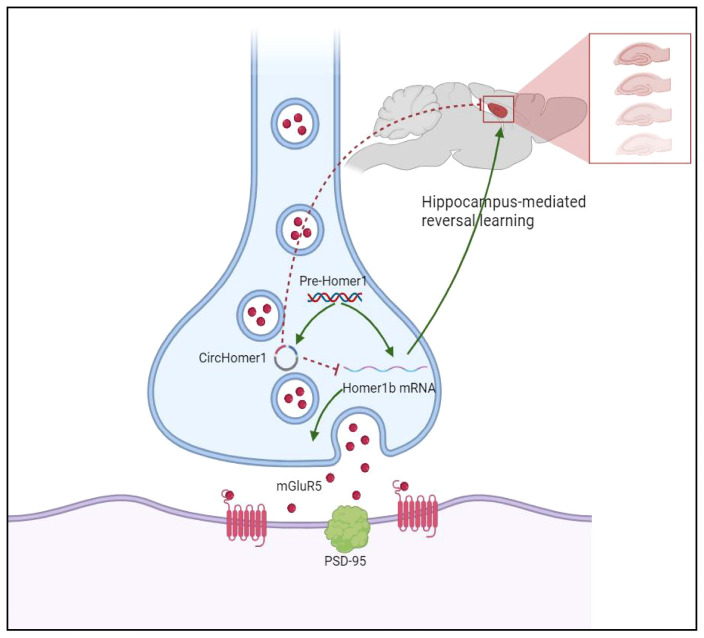
Illustration of a synapse showing the molecular pathway involving Pre-Homer1, CircHomer1, and Homer1b mRNA, with arrows indicating release and interaction with mGluR5 and PSD-95, linked to hippocampus-mediated reversal learning in the brain.

## Introduction

Postoperative delirium (POD) is a severe postoperative complication and also a significant public health challenge associated with global aging (Oh et al., 2017; Swarbrick and Partridge, 2022). It is significantly correlated with adverse postoperative outcomes, such as prolonged hospital stays and increased readmission rates (Goldberg et al., 2020; Hshieh et al., 2015). POD has an acute onset and a variable course, with patients exhibiting varying degrees of consciousness, poor cognitive ability, memory impairment, disorientation, and sleep-wake cycle disturbances (Jin et al., 2020). What’s more, if patients experience postoperative delirium, their long-term cognitive function will significantly decline (Needham et al., 2017), and the risk of developing Alzheimer’s disease will also increase (Inouye et al., 2016; Koster et al., 2012). These conditions not only severely affect patients’ learning, life, and social activities but also impose a huge economic burden on society (Yang et al., 2020). However, the current prevention and treatment options for POD are limited (Safavynia et al., 2018), and there is an urgent need for new therapeutic approaches to prevent or delay the continuous deterioration of brain function.

Metabotropic glutamate receptor 5 (mGluR5) is widely distributed in the nervous system and is extensively involved in synaptic plasticity, learning, and memory (Abd-Elrahman et al., 2020; Morató et al., 2018; Schorova et al., 2019; Wang et al., 2020; Zantomio et al., 2015). It also plays an important regulatory role in various diseases such as Fragile X syndrome, schizophrenia, Parkinson’s disease, anxiety disorders, and chronic pain (Danjo et al., 2022; Li et al., 2023; Matosin et al., 2017; Stoppel et al., 2021; Zhang et al., 2019). CHPG (2-chloro-5-hydroxyphenylglycine) is a selective mGluR5 agonist, and 6-methyl-2-(phenylethynyl)pyridine (MPEP) is a potent, selective, non-competitive, and systemically active mGluR5 receptor antagonist (Domenici et al., 2005; Qiu et al., 2015). In addition to these two agonists and inhibitors, Homer1 can also affect the distribution of mGluR5 on different cells and regulate the interaction between mGluR5 and downstream molecules (Potschka et al., 2002; Qiu et al., 2015). Therefore, regulating the expression of Homer1 to modulate the distribution of mGluR5 on the cell membrane and downstream signal transduction may be the key to the involvement of the Homer1/mGluR5 signaling pathway in the regulation of learning and memory functions.

Homer1 protein is an important cytoskeletal protein and a component of the postsynaptic density (PSD), deeply involved in the functional regulation of synaptic plasticity, learning, and memory. It has been reported to be involved in various cognitive disorders, such as schizophrenia (SCZ), bipolar disorder (BD), and traumatic brain injury (TBI; de Bartolomeis et al., 2022; Wagner et al., 2013; Zimmerman et al., 2020). Previous studies have shown that Homer1 protein can be divided into short Homer (Homer1a) and long Homer (Homer1b/c) based on the presence or absence of a coiled-coil structure at the C-terminus. Homer1a is usually not expressed or rarely expressed, while Homer1b/c is stably expressed throughout the body (de Bartolomeis et al., 2022; Fei et al., 2014; Wu et al., 2022; Yoon et al., 2021). In our previous study, we used circular RNA chip technology to analyze the differential expression profile of circRNA in the hippocampus of 12–14 month-old POD-like mice and control mice, and found that circHomer1 was specifically and unexpectedly increased in the glutamatergic synaptic system (Ran et al., 2022).

CircHomer1 is derived from the transcription product of Homer1 protein and is highly expressed in the frontal cortex. In the prefrontal cortex (PFC) of patients with schizophrenia (SCZ) and bipolar disorder (BD), as well as in induced pluripotent stem cell-derived neuronal cultures, its expression is significantly reduced. Hafez et al. (2022) reported that circHomer1 is negatively correlated with the levels of Homer1b mRNA homologs in the orbitofrontal cortex (OFC) and stem cell-derived neuronal cultures of patients with mental disorders. Recent studies have shown that the vCA1 region is closely related to conditioned fear memory and learning (Hong and Kaang, 2022; Jimenez et al., 2020; Kim and Cho, 2020).

Based on the above analysis, we speculate that circHomer1 in the vCA1 region may inhibit the expression of Homer1b, reduce its binding with downstream mGluR5, and may modulate Homer1b/mGluR5-associated signaling and contribute to POD-like postoperative cognitive impairment.

## Materials and methods

### siRNA validation and screening

For the *in vitro* culture of hippocampal neurons, growth was observed every day. Then, the siRNAs and control reagents were added during the rapid cell proliferation stage. The initial transfection concentration was 30 nM, and the detection time after transfection was 24 h after transfection. After 72 h, the expression levels of circHomer1 and Homer1b were measured using qRT–PCR, and the siRNA with the best interference effect was verified. After modification and optimization, the selected siRNA was injected into the CA1 region of the ventral hippocampus of the mice. The design and synthesis of the siRNAs were performed by RiboBio, and the designed sequences are shown in Supplementary Table 2.

### siRNA interference in cells

The experiment was conducted according to the group and reagent manufacturer’s instructions (RiboFECT™ CP Reagent, Shen zhen, China). The transfected siRNA was added to cells in a 96-well Petri dish at a concentration of 30 nmol (Ribobiological RN:R10043.8). Three replicate wells were set for each transfected sample during transfection. The number of cells inoculated in each well was as consistent as possible so that the inoculated cells were evenly distributed on the surface of each well (The C57BL/6 mouse hippocampal neurons were kindly provided by the research group of Professor Wang Xuefeng from the Key Laboratory of Neurology and the Department of Neurology, The First Affiliated Hospital of Chongqing Medical University).

### Modified siRNAs used in animal experiments

Due to the high requirements for siRNA stability in animal experiments, although unmodified siRNAs can also be tested in animal experiments, the industry still recommends the use of modified siRNAs for injection. At present, Chol, OMe and PS are mostly used to modify target siRNAs. The experiment was conducted using the Ribobio 5′ Chol + 2′ OMe modification scheme, and the modified siRNA was used for subsequent animal experiments.

### Animals

This experiment was performed in accordance with the Guidelines for the Care and Use of Experimental Animals and approved by the Animal Ethics Committee of the First Affiliated Hospital of Chongqing Medical University. Eighty 12–14 month-old male C57BL/6J mice were purchased from the Animal Experimental Center of Chongqing Medical University (Chongqing, China). The mice weighed 30–35 g, were housed in groups for 1 week, and fed regular rodent chow (temperature 25 °C, humidity 55%, 12-h light/dark cycle).

### Animal model of POD-like postoperative neurocognitive impairment

Intramedullary nail fixation of the tibial fracture in the left hind leg was performed with isoflurane for anesthesia (2% isoflurane for induction, 1.5% isoflurane for maintenance; Baxter International, Deerfield, IL, USA) and subcutaneous Butonofi for analgesia (0.1 mg/kg, Jiangsu Hengrui Pharmaceutical Co., Ltd., Lianyungang, China). All mice were placed in the supine position after successful anesthesia. The left hind leg was disinfected with povidone iodine, and a lateral incision of approximately 1 cm was made in the upper tibia. An osteotomy was performed in the middle of the tibia. A sterile steel needle with a diameter of 0.38 mm was inserted into the tibial pulp cavity to link the broken ends. The end of the needle was cut off at the level of the tibial plateau. After flushing, the wound was closed with 5–0 nylon sutures. Then, maintenance anesthesia was stopped, and the mice were returned to the cage to awaken naturally. All procedures were completed within 15 min. Mice from the control group were administered the same doses of anesthesia and analgesia to control for the effects of bias. The number of mice in each group was 10 (*N* = 10). All mice survived until the third day after surgery (survival rate 100%), and tissue collection was performed after the behavioral tests were completed.

### Behavioral tests

#### Trace fear conditioning test (TFC)

Our experimental design was slightly modified from previous studies (Chong et al., 2023; Li et al., 2019; Wei et al., 2017; Xiang et al., 2019). All mice were placed in the test chamber on the preoperative day and 30 min before surgery (9:00 am) and allowed 3 min of free exploration. All mice were subjected to three rounds of sound (5,000 Hz, 80 dB, and 30 s) and electric shock stimulation (0.8 mA, 2 s). The shock followed the sound stimulus with a 58-s delay between the two stimuli. A Panlab fear conditioning test device was used to measure the freezing time of the mice in both the contextual and cued tests, and all the data were processed with PACKWIN v2.0.05 (Šmidák et al., 2016; Tishkina et al., 2016).

On postoperative Day 3, contextual and cued assessments were completed at 9:00 a.m. Mice were allowed to explore for 5 min without being stimulated by sound or electric shock; 2 h later, the mice were placed in an environment different from the preoperative training setting (internal color change). After a 3-min exploration, the mice were subjected to identical sound stimulation (5,000 Hz, 80 dB, and 30 s) but no electric shock. The interval between the two stimuli was still 58 s. The box was cleaned with alcohol to ensure that the odor of the previous mouse did not affect the behavior of the next animal.

#### Open field test (OFT)

The OFT was used to determine the activity of the experimental animals. All mice were placed in the central region of the experimental apparatus (45 × 45 × 60 cm). A digital camera was used to record the activity data (how long the mice stayed in the center square and the edge zone and the distance traveled by mice in each area) of the mice during the 3-min test, and the data were analyzed using SPSS 25.0.

### Intracerebral injections of lentivirus

All procedures were performed using aseptic techniques and isoflurane gas anesthesia. The bilateral ventral hippocampus was injected using a stereotaxic framework (Kopf Instruments, Tujunga, CA), during which the subject was maintained under isoflurane anesthesia. The injection was performed with a 101Hamilton syringe fitted with a custom-beveled 32-gauge needle (Hamilton). The injection speed was precisely adjusted. The needle was then left in place for5 min before being removed from the brain. The coordinates for ventral CA1 injection of si-Homer1 and si-circHomer1 into mice were as follows: temporal site (AP = −2.5, Lat = −3.0, DV = 4.0).

### RNA extraction

On the third day after surgery, hippocampal tissues were extracted from all groups of mice and immediately stored at −80 °C. TRIzol reagent (Invitrogen, Carlsbad, CA, USA) was used to extract total RNA from both sets of samples according to the manufacturer’s instructions.

### Protein extraction and Western blot analysis

The expression of target proteins was determined using Western blotting. The specific method was as follows: an SDS–PAGE gel with a lower gel concentration of 10% and an upper gel concentration of 5% was prepared. According to the results of protein quantification, 40 μg of protein from each sample was loaded into each well, and the prestained marker was added to one lane. After electrophoresis and electrorevolution, the PVDF membrane was removed with tweezers and washed 3 times with TBST. The PVDF membrane was then placed in rapid sealing solution, incubated with shaking for 20 min at room temperature, and rinsed with TBST 3 times. Rabbit anti-mGluR5 and PSD-95 antibodies (1:1000, Abcam, USA) and mouse anti-β-actin antibodies (1:1000, Abcam, USA) were added. The mixture was incubated with the membrane at 4 °C overnight. After the overnight incubation, the membranes were rinsed with TBST (5 min/wash, 3 times) to remove unbound primary antibodies. Goat anti-rabbit IgG (1:2500, Abcam, USA) and goat anti-mouse IgG (1:2500, Abcam, USA) were added to the membranes and incubated at room temperature for 1 h. The unbound secondary antibodies were removed by rinses with TBST (5 min/wash, 4 washes), approximately 100 μl of the prepared ECL developer was added to the proteins on the surface of the PVDF membrane, and the membrane was then exposed on a gel imager. The images were captured and analyzed using Bio-Rad quantitative analysis software.

### Quantitative real-time PCR

The TAKARA PrimeScript RT reagent Kit TRR037A reverse transcription–polymerase chain reaction system (TAKAR Japan) was used for quantitative real-time polymerase chain reaction (qRT–PCR), and the specific primer pairs used in the study are listed in Supplementary Table 1.

### Immunohistochemistry (IHC)

Immunohistochemical staining was performed as described below. Paraffin-embedded specimens were sectioned at 5 μm, deparaffinized, and hydrated. The sections were then incubated with 3% H_2_O_2_ for 10 min and rinsed with phosphate-buffered saline (PBS). Primary antibodies against mGluR5 were used. The sections were then washed and incubated with secondary antibodies at room temperature for 30 min. The sections were observed under an optical microscope.

### Golgi staining

An FD Rapid Golgi Stain Kit (FD Neurotechnologies) was used for Golgi–Cox staining. After euthanasia and dissection (All experimental animals were handled in strict accordance with the ARRIVE guidelines and the American Veterinary Medical Association (AVMA) Guidelines for the Euthanasia of Animals: 2020 Edition. Mice were deeply anesthetized with an intraperitoneal injection of pentobarbital sodium (50 mg/kg). Once the absence of a pedal withdrawal reflex confirmed a surgical plane of anesthesia, animals were humanely euthanized by cervical dislocation. Death was finally confirmed by the complete cessation of respiration and cardiac activity before any tissue was harvested), the brain tissues were impregnated in equal volumes of Solutions A and B containing mercuric chloride, potassium dichromate, and potassium chromate and incubated at room temperature for 14 days. The impregnation solution was changed every 24 h. On Day 15, the brain samples were placed in Solution C and incubated at 4 °C for 48 h. As previously stated, the solution was replaced every 24 h. Sagittal dissection under cryogenic conditions was employed to produce sections with a thickness of 100 μm before the cut sections were mounted on gelatin-coated microscope slides with Solution C and stained.

### Electron microscopy

The dendrite analysis was limited to single neurons from the vCA1. Z-stack images of these neurons were acquired using an upright microscope (STE system; Hitachi). ImageJ software (NIH) with the NeuronJ plugin was used to generate dendritic branch traces and to determine the length and number of dendritic branches and spines. We used ImageJ software (Sholl analysis plugin) to count the number of crossings of concentric circles by dendrites beginning from the soma to examine neuronal arborization.

### Statistical analysis

Statistical analyses were performed using GraphPad Prism 8.3.0 (GraphPad, La Jolla, CA) and SPSS 25.0. For the comparison of measurement data between different groups, independent samples Student’s *t*-test or the Wilcoxon-Mann-Whitney test was used. Normally distributed data were expressed as mean ± standard deviation (SD), while non-normally distributed data were expressed as median [interquartile range]. Analysis of variance (ANOVA) was used for comparisons between groups, and F/p value were provided in each part of results. LSD-t test was used for comparisons between groups, and bilateral *P* < 0.05 was considered to indicate statistical significance.

## Results

### Determination of circHomer1 and Homer1b siRNA transfection sequences

**FIGURE 1:**
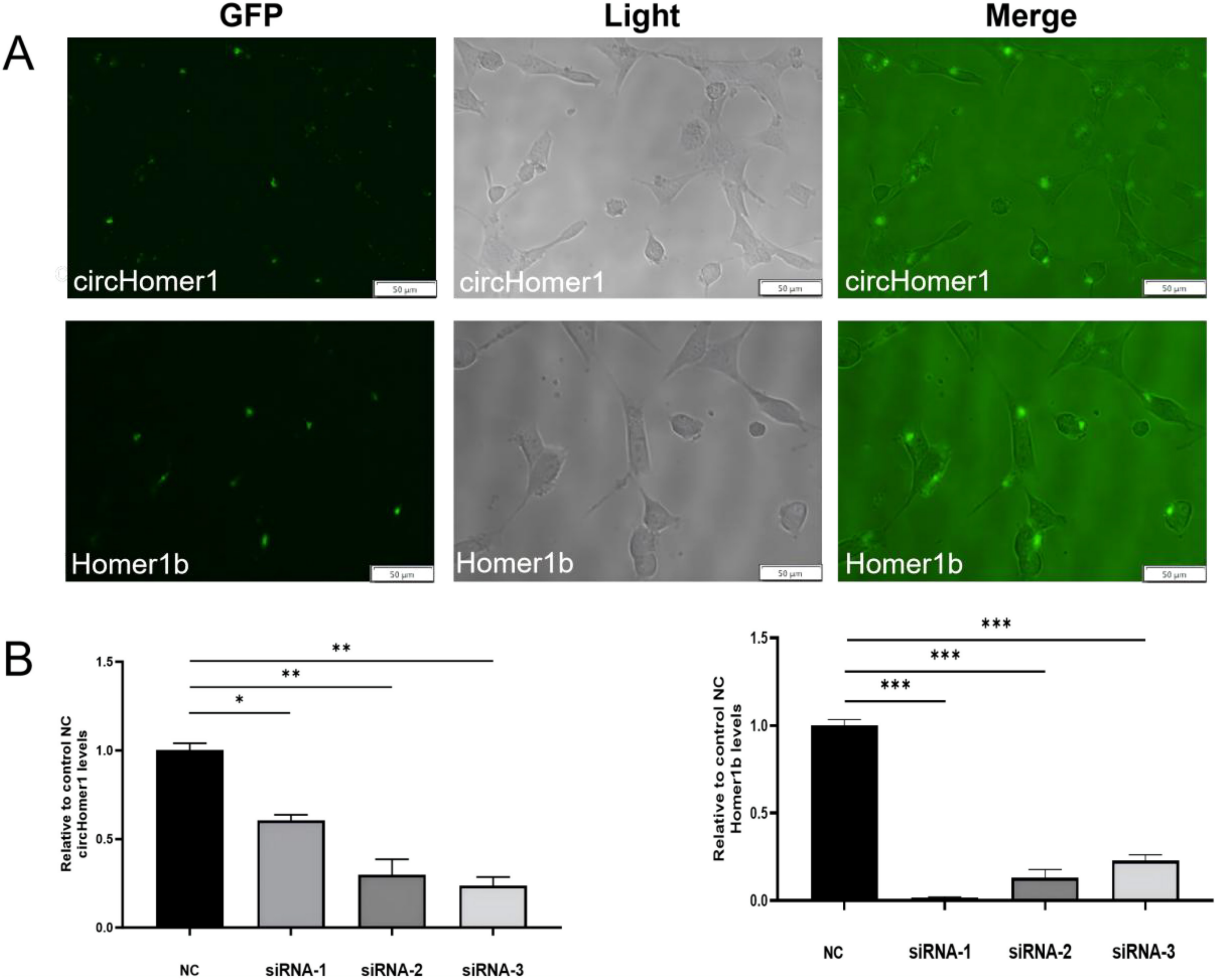
siRNA transfection experimental results. **(A)** Fluorescence images of hippocampal neurons transfected with siRNA. **(B)** Expression levels of circHomer1 and Homer1b measured using qRT–PCR (*F* = 273.8, *P* < 0.0001, *F* = 1271, *P* < 0.0001). Data from multiple groups were analyzed using analysis of variance (ANOVA), and the significance of differences between multiple groups was determined using LSD-t test. IntDen refers to integrated density. Images were analyzed using ImageJ software. *P < 0.05, **P < 0.01, ***P < 0.001, Number of cell wells used in each group (*N* = 5).

Cellular experiments demonstrated that upon the addition of the corresponding siRNAs to the culture dishes of mouse hippocampal neurons, the siRNAs were taken up by the cells, as observed under a fluorescence microscope (Figure 1A). The expression of circHomer1 and Homer1b was detected using PCR, and the siRNAs were found to reduce the expression levels of the target genes (Figure 1B) (*F* = 273.8, *P* < 0.0001, *F* = 1271, *P* < 0.0001). siRNA-3 was selected as the experimental sequence for interfering with circHomer1, and siRNA-1 was chosen for interfering with Homer1b. Sequence information was provided in Supplementary Table 2.

### Tibial fractures can lead to cognitive dysfunction in elderly mice.

**FIGURE 2:**
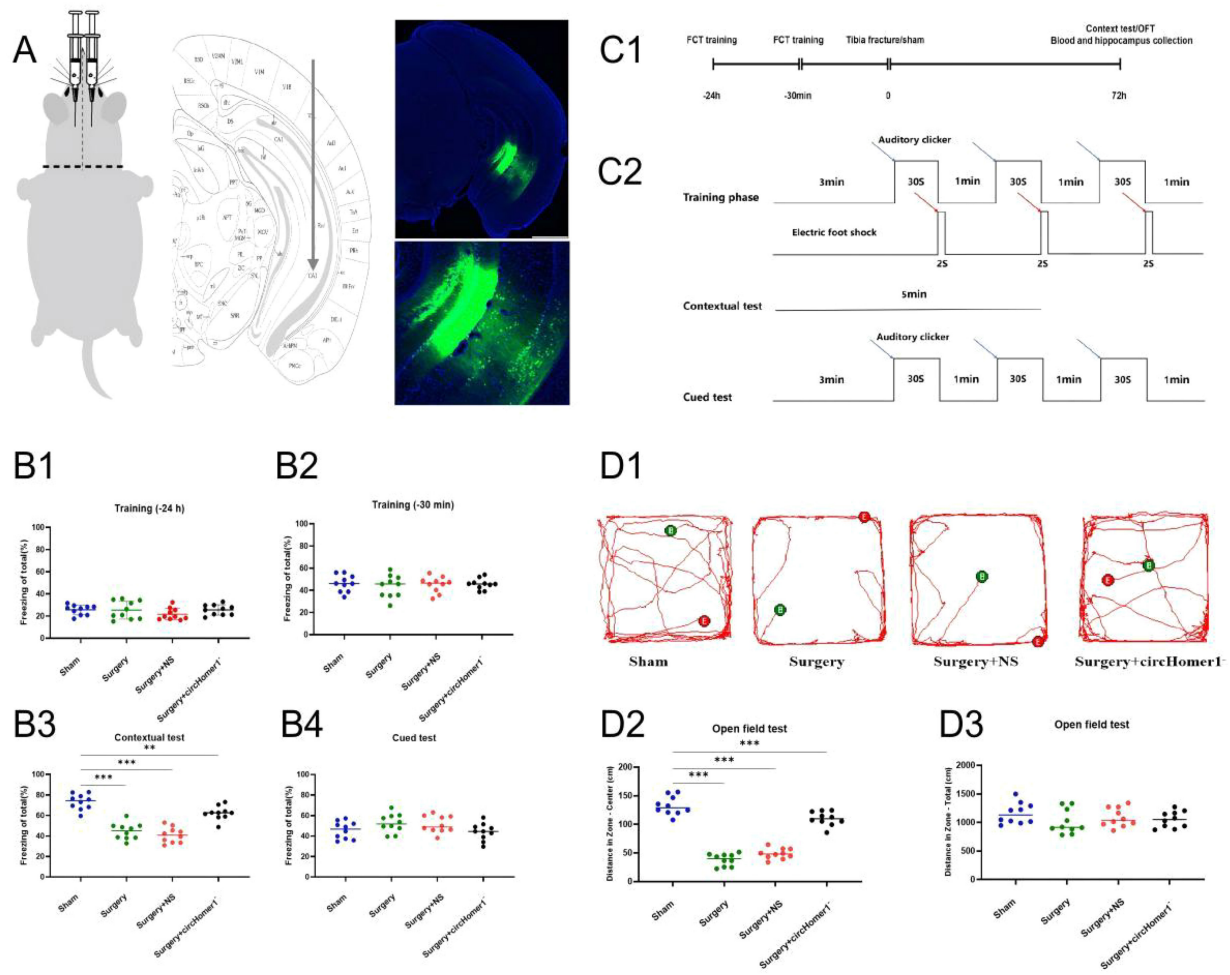
Cognitive impairment in elderly mice induced by tibial fracture. **(A)** Site of administration, neurons in the vCA1 region of mice were infected with green fluorescent virus. **(B)** TFC training, contextual test, and cue test. **(C)** Experimental process and TFC experimental design. **(D)** Mouse trajectories in the open field; the starting and ending points are indicated in green and red, respectively. *P < 0.05, **P < 0.01, ***P < 0.001, Number of animals used in each group (*N* = 6-8).

There were no significant differences in the freezing time of the four groups of mice during TFC training one day before surgery and 30 min before surgery (*P* > 0.05), as shown in Figures 2B1, B2. In the contextual memory test on the third day after surgery, the freezing time of the mice after surgery was significantly shorter than that of the control group (*F* = 42.34, *P* < 0.0001) (Figure 2B3), but there was no significant difference in the freezing time between the two groups in the cue test (*P* > 0.05) (Figure 2B4). In the open field test (OFT) on the third day after surgery, there were no significant differences in the walking distance of the four groups of mice (*P* > 0.05) (Figure 2D3), but the mice after surgery spent significantly less time in the center area than the other groups (*F* = 84.31, *P* < 0.0001) (Figure 2D2). These data indicate that cognitive dysfunction occurred in mice on the third day after tibial fracture intramedullary nail fixation surgery. Interfering with the expression of circHomer1 can rescue the freezing time in the contextual fear memory test and the time spent in the center area of the open field test (Figures 2B3, D2), indicating that interfering with the expression of circHomer1 can rescue cognitive dysfunction in mice.

### Interfering with circHomer1 can reduce neuronal shrinkage in the vCA1 region of elderly mice and increase the expression of mGluR5 protein

**FIGURE 3:**
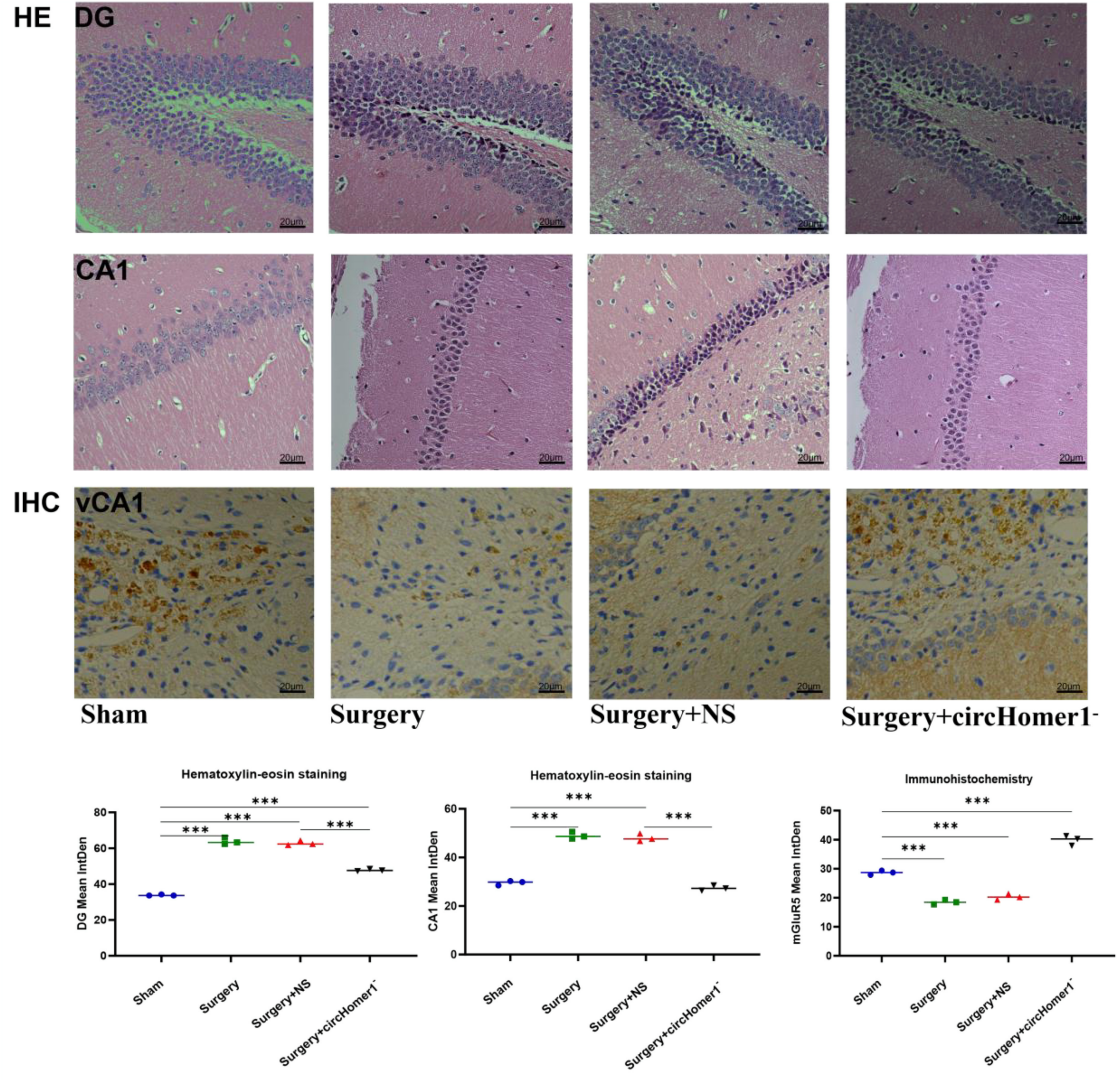
Hippocampal HE staining and immunohistochemistry. HE refers to Hematoxylin and Eosin staining, and IHC refers to Immunohistochemistry. DG, CA1, and vCA1 refer to the hippocampal DG, CA1, and ventral CA1 regions, respectively. IntDen refers to integrated density. *P < 0.05, **P < 0.01, ***P < 0.001, Number of animals used in each group (*N* = 3-8).

Hematoxylin and Eosin staining showed that neurons in the hippocampal CA1 and DG regions of the surgery group mice were significantly contracted, with the average IntDen in the DG region being significantly higher than that in the sham surgery group (*F* = 328.8, *P* < 0.0001), and similarly, the average IntDen in the CA1 region was also significantly higher than that in the sham surgery group (*F* = 559.2, *P* < 0.0001) (Figure 3). Further analysis indicated that the average density in the vCA1 region of the circHomer1 interference group was lower than that in the surgery group, while this improvement was not significant in the DG region. Immunohichestomistry showed that the average IntDen of mGluR5 in the surgery group and the surgery **+** NS group was significantly lower than that in the sham surgery group (*F* = 224.6, *P* < 0.0001), while the expression level of mGluR5 in the circHomer1 interference group was higher than that in the surgery group. Combining the behavioral data analysis from Figure 2, the cognitive impairment observed in mice on the third day after tibial fracture intramedullary nail fixation surgery may be related to the reduced expression of mGluR5, and interfering with the expression of circHomer1 can rescue the degree of cognitive dysfunction.

### Interfering with circHomer1 can reduce the expression of circHomer1 and Homer1a in the vCA1 region of elderly mice, while promoting the expression of Homer1b

**FIGURE 4:**
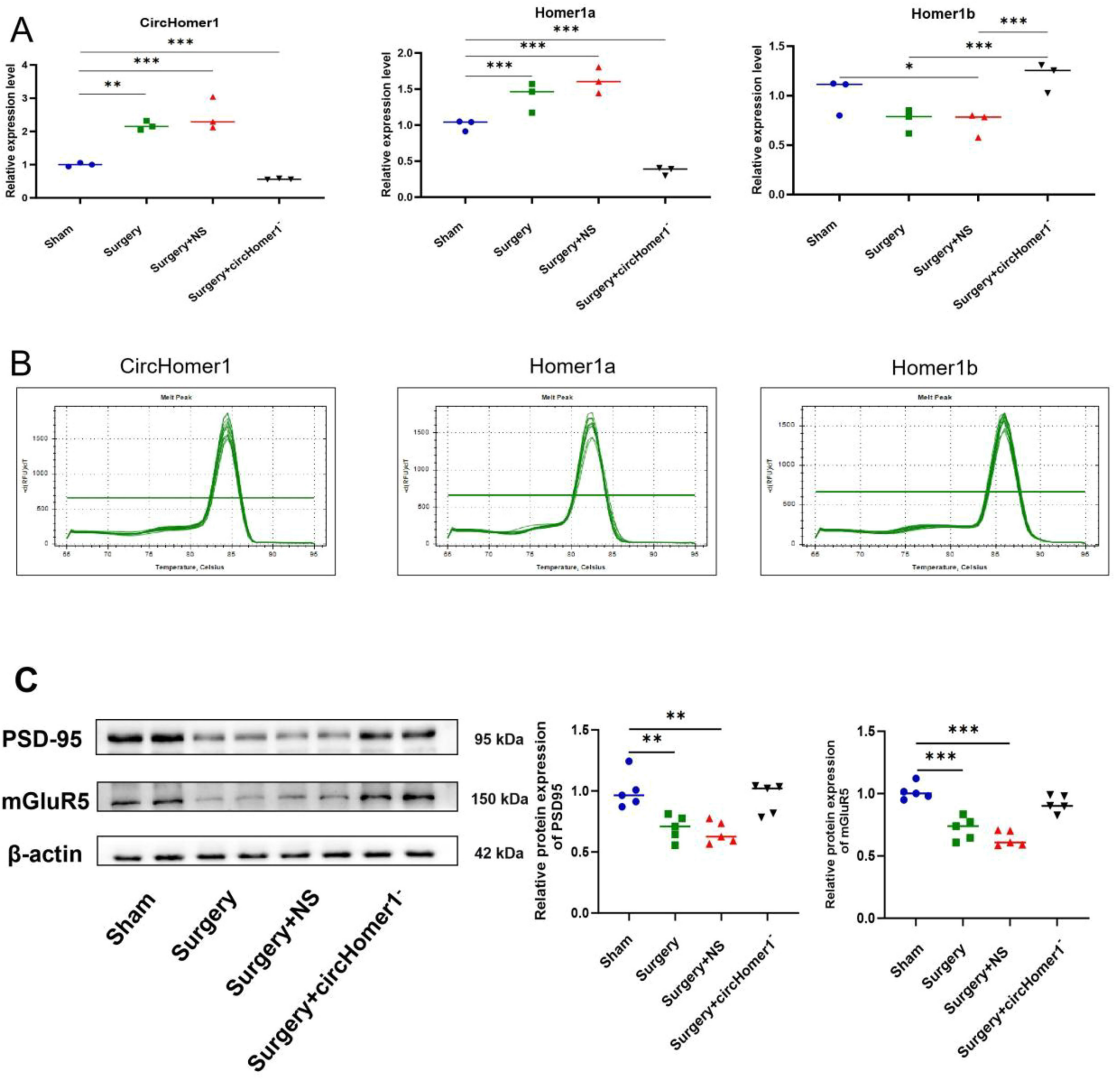
PCR and Western blot analysis of target molecules in the hippocampus. **(A)** Expression levels of circHomer1, Homer1a, and Homer1b mRNA. **(B)** PCR melting curves indicating good specificity of the primers. **(C)** Western blot analysis. *P < 0.05, **P < 0.01, ***P < 0.001, Number of animals used in each group (*N* = 3-8).

Polymerase chain reaction analysis revealed that, compared to the control group, the expression levels of circHomer1 and Homer1a were significantly increased in the surgery and surgery **+** saline groups (*F* = 49.91, *P* = 0.0001; *F* = 57.92, *P* < 0.0001), while these levels were significantly reduced in the circHomer1 interference group (Figures 4A,B). The expression levels of Homer1b were significantly decreased in the surgery and surgery **+** saline groups (*F* = 71.52, *P* < 0.0001), but were significantly increased in the circHomer1 interference group. Western blot experiments showed that the expression levels of mGluR5 and PSD-95 proteins were significantly decreased in the surgery and surgery **+** saline groups, but were significantly increased in the circHomer1 interference group (Figure 4C). These data suggest that elevated levels of circHomer1 and Homer1a may be risk factors for POD-like postoperative neurocognitive impairment, while increased expression of Homer1b may serve as a protective factor against POD-like postoperative neurocognitive impairment progression, potentially achieved through increased expression of mGluR5 and PSD-95 proteins.

### Interfering with circHomer1 can reduce dendritic spine damage in the vCA1 region of elderly mice

**FIGURE 5:**
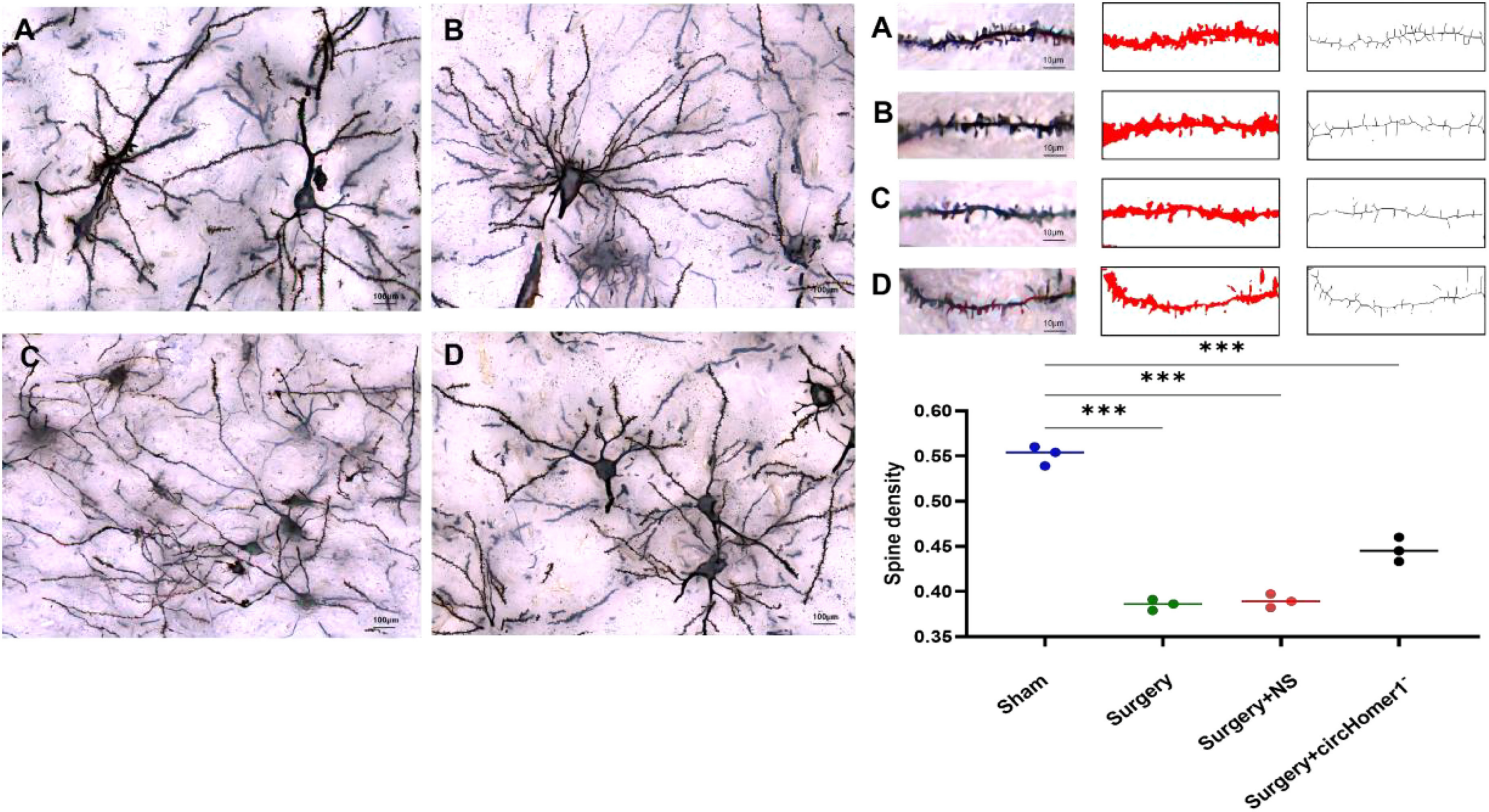
Golgi staining of the vCA1 region in the hippocampus of elderly mice. **(A)** Sham surgery group; **(B)** Surgery group; **(C)** Surgery + saline group; **(D)** Surgery + circHomer1 interference group. *P < 0.05, **P < 0.01, ***P < 0.001, Number of animals used in each group (*N* = 5–8).

Golgi staining showed that, compared to the control group (Figure 5A), the dendritic spine density was significantly reduced in the surgery group (Figure 5B) and the surgery + saline group (Figure 5C) (*F* = 182.1, *P* < 0.0001), while the reduction was mild in the circHomer1 interference group (Figure 5D). This indicates that interfering with circHomer1 can mitigate the dendritic spine damage in the vCA1 region of elderly mice.

### Interfering with circHomer1 can alleviate neuronal myelin damage in the vCA1 region of elderly mice

**FIGURE 6:**
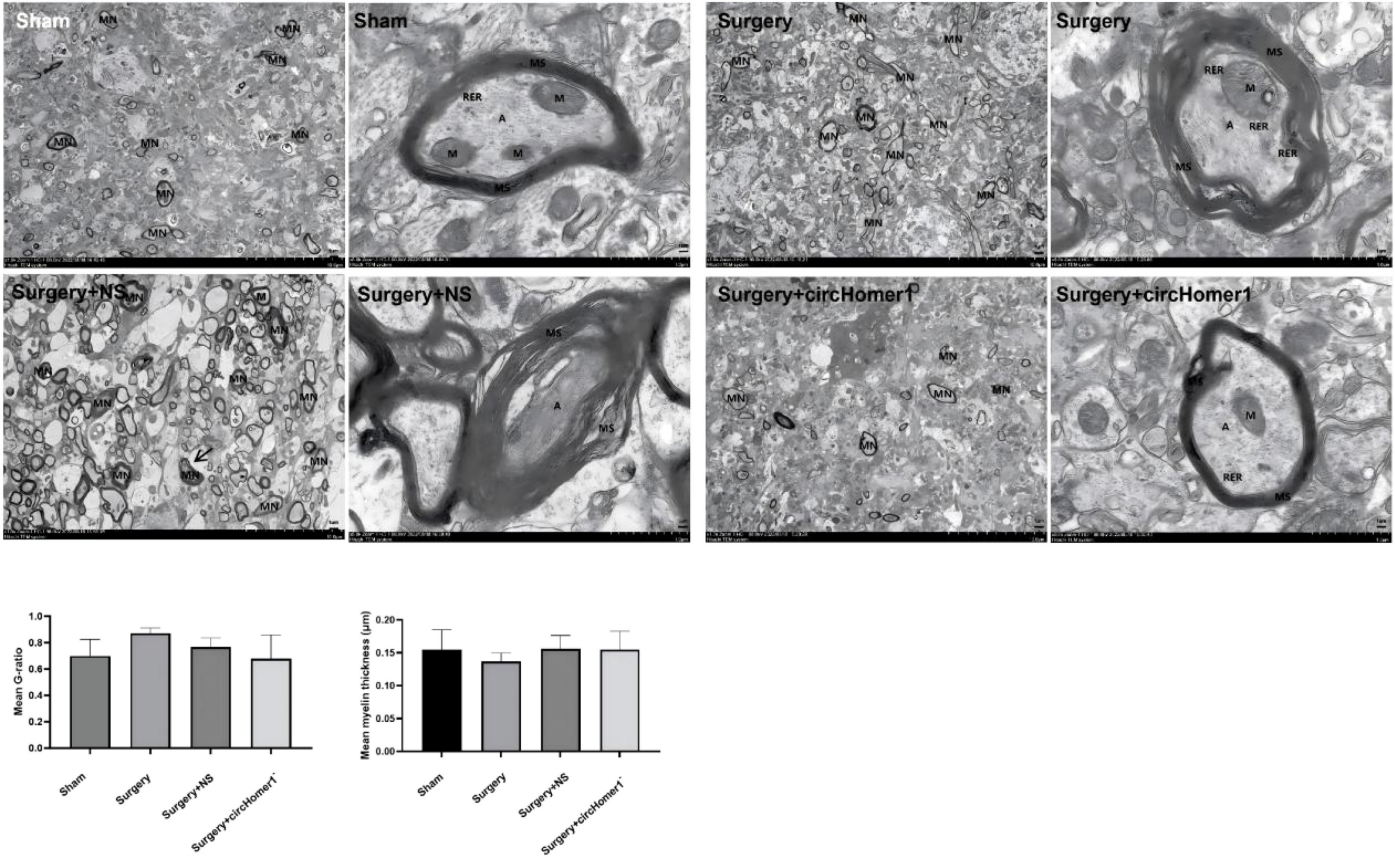
Transmission electron microscopy images of the vCA1 region in the hippocampus of elderly mice. MN indicates myelinated nerve, MS indicates myelin sheath, A indicates axon M indicates mitochondria, and RER indicates rough endoplasmic reticulum. Myelin thickness (μm), diameter of myelinated nerve fibers (μm), and axon diameter (μm) were measured using Image-Pro Plus 6.0 software, and the G-ratio was calculated. *P < 0.05, **P < 0.01, ***P < 0.001, Number of animals used in each group (*N* = 6-8).

Compared to the damage in the control group, the myelin nerve damage was more severe in the surgery group and the surgery + saline group. In the surgery group alone, many layers showed loose structure, with slight axonal atrophy locally, mild edema under the axolemma, and mild mitochondrial swelling. In the surgery + saline group, some layers were loose and uneven in thickness, with axons being compressed and some axons showing obvious demyelination (indicated by black arrows), as well as local axonal atrophy, narrowing, and edema under the axolemma. In the surgery + circHomer1 group, the degree of myelin nerve damage was relatively mild, with most nerves being uniform in thickness, clear in structure, and occasionally slightly dispersed. No obvious axonal atrophy was observed, and there was only mild swelling of mitochondria and rough endoplasmic reticulum. There were no significant differences between groups in terms of myelin thickness, diameter of myelinated nerve fibers, axon diameter, or G-ratio (average G-ratio: *F* = 1.105, *P* = 0.446; average myelin thickness *F* = 0.152, *P* = 0.923) (Figure 6).

### Overexpression or interference of Homer1b can increase or suppress the expression of mGluR5 and PSD-95 proteins, thereby improving or impairing cognitive function

**FIGURE 7:**
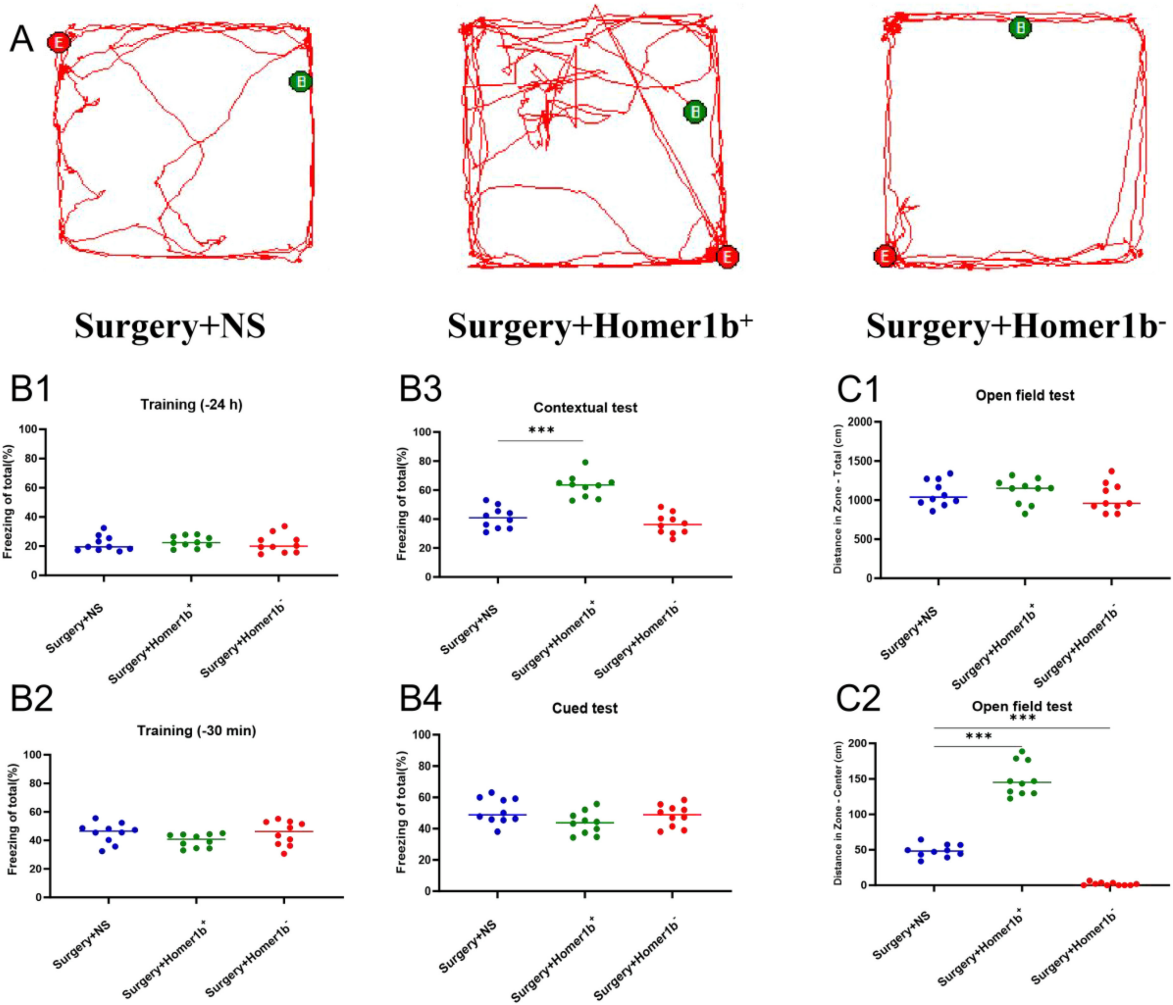
Behavioral data from open field and contextual fear conditioning experiments. **(A)** Schematic of the open field test. **(B)** Schematic of the fear conditioning test. **(C)** Graphs of data from the open field test. *P < 0.05, **P < 0.01, ***P < 0.001. Number of animals used in each group (*N* = 7-8).

Behavioral data from the open field and contextual fear conditioning experiments showed differences in the behavior of mice following overexpression or interference of Homer1b (Figure 7A). Specifically, mice with overexpression of Homer1b exhibited enhanced memory in the contextual test, while mice with interference of Homer1b showed significantly impaired memory (Figure 7B3). Additionally, the open field test revealed no significant differences in the walking distance between the two groups, but mice with overexpression of Homer1b spent significantly more time exploring the center area compared to those with Homer1b interference (Figures 7C1, C2) (*F* = 264.7, *P* < 0.0001). These data suggest that the expression level of Homer1b is closely related to the progression of POD-like postoperative neurocognitive impairment.

**FIGURE 8:**
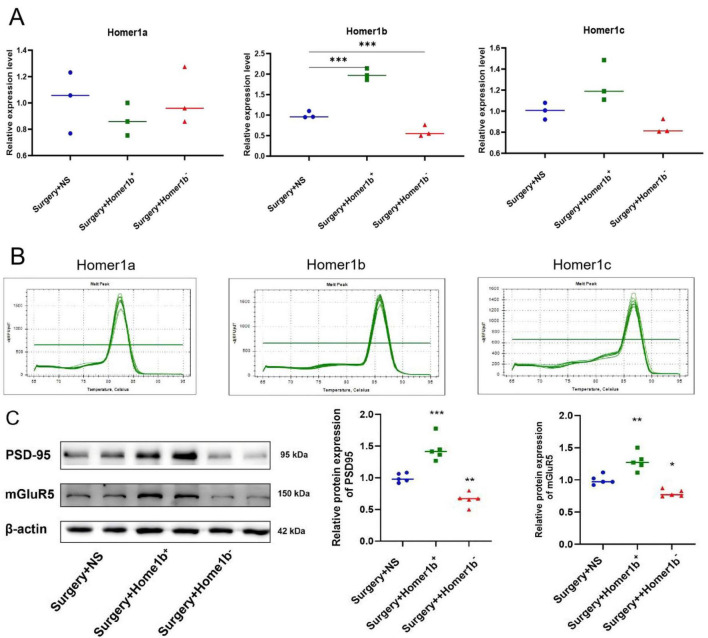
PCR and Western blot analysis of target molecules in the hippocampus. **(A)** Expression levels of Homer1a, Homer1b, and Homer1c mRNA. **(B)** PCR melting curves indicating good specificity of the primers. **(C)** Western blot analysis. *P < 0.05, **P < 0.01, ***P < 0.001, Number of animals used in each group (*N* = 3-8).

PCR analysis revealed that, compared to the control group, the expression levels of Homer1a and Homer1c did not show significant differences following overexpression or interference of Homer1b (Figures 8A,B). However, the expression level of Homer1b itself was significantly increased or decreased, respectively. Western blot analysis showed that the expression levels of mGluR5 and PSD-95 proteins were significantly increased following overexpression of Homer1b and significantly decreased following interference of Homer1b (Figure 8C). These data suggest that Homer1b influences the progression of POD-like postoperative neurocognitive impairment by regulating the expression of mGluR5 and PSD-95 proteins.

### Activating or interferencing the function of mGluR5 receptors can rescue or impair cognitive function in elderly mice

**FIGURE 9:**
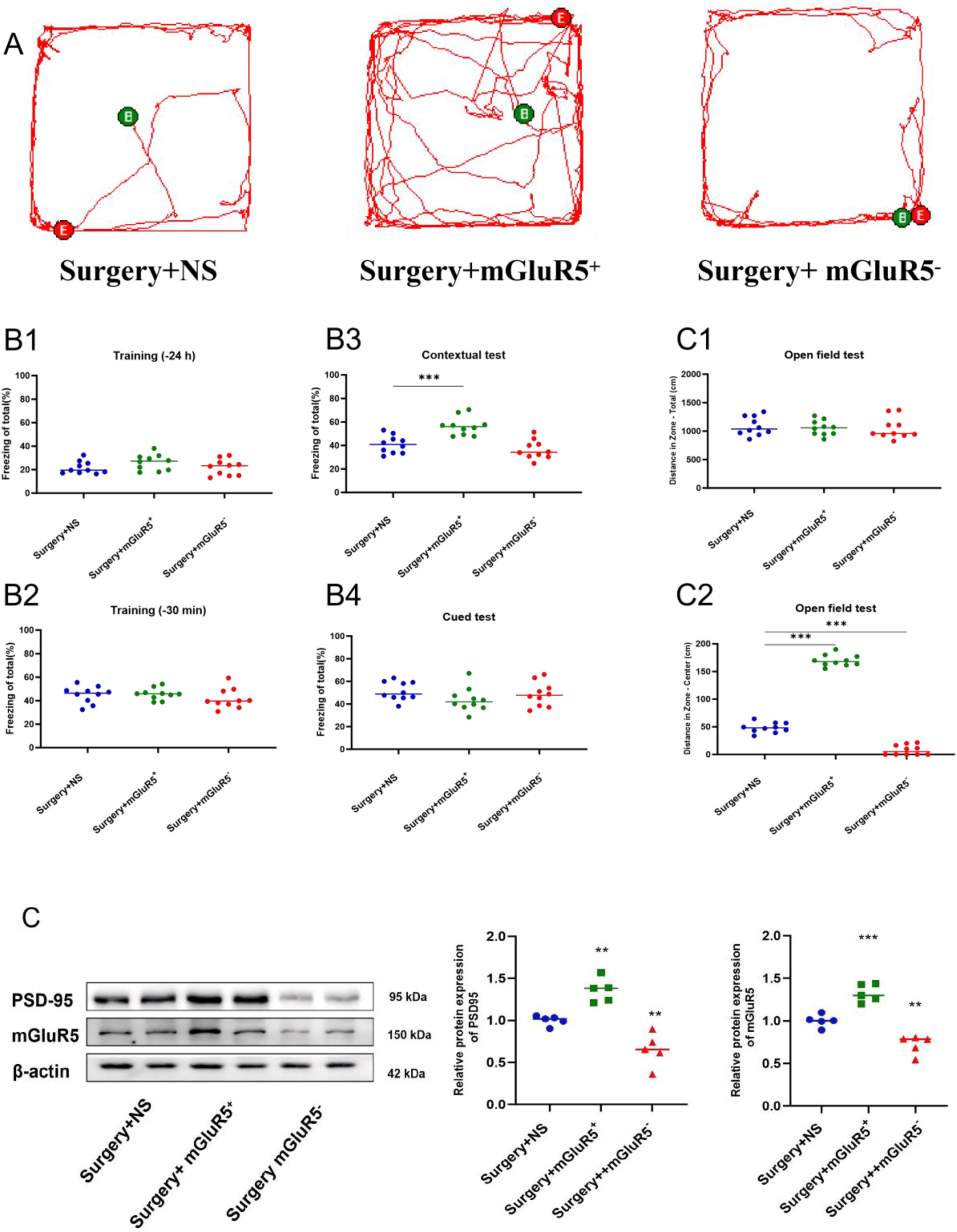
Behavioral changes following mGluR5 overexpression and interferencing. **(A)** Schematic of the open field test. **(B)** Schematic of the fear conditioning test. **(C)** Graphs of data from the open field test. *P < 0.05, **P < 0.01, ***P < 0.001. Number of animals used per group (*N* = 5–8).

The expression levels of mGluR5 and PSD-95 proteins were significantly increased following overexpression of mGluR5 and significantly decreased following silencing of mGluR5 (Figure 9C). Behavioral data from fear conditioning and open field experiments showed that mice with overexpression of mGluR5 exhibited enhanced memory in the contextual test, while mice with silencing of mGluR5 showed significantly impaired memory (Figures 9A,B3). Additionally, the open field test revealed that mice with overexpression of mGluR5 spent significantly more time exploring the center area compared to those with silencing of mGluR5, although no significant differences were observed in other aspects (Figures 9C1, 9C2) (*F* = 776.2, *P* < 0.0001). These data indicate that the expression level of mGluR5 is also closely related to the progression of POD-like postoperative neurocognitive impairment.

## Discussion

Our study revealed that in the vCA1 region of elderly mice, circHomer1 inhibits the expression of Homer1b through the Homer1/mGluR5 signaling pathway, thereby promoting the progression of POD-like postoperative neurocognitive impairment. In this study, we observed the interaction between circHomer1 (transcribed from the intronic sequence of the Homer1 gene) and linear Homer1b mRNA in the hippocampus of POD-like mice, an interaction that can regulate the degree of cognitive impairment in elderly mice following tibial fracture.

To further substantiate that circHomer1 interference alleviates postoperative cognitive impairment in elderly mice, we provide the following evidence: HE Staining Observations: We observed that hippocampal neurons in the surgical group of mice contraction. The phenomenon of neuronal contraction was highly correlated with cognitive dysfunction, which became one of the important pieces of evidence for the occurrence of POD-like postoperative neurocognitive impairment and was similar to the previous research results on cognitive impairment (Chen et al., 2021; Ran et al., 2022). In contrast, the circHomer1 interference group showed reduced neuronal tangles and shrinkage. Immunohistochemistry for mGluR5 Protein Expression: We determined the expression of mGluR5 protein in the vCA1 region using immunohistochemistry and found that the expression levels were significantly reduced in POD-like mice induced by surgery. In the circHomer1 interference group, the mGluR5 levels were only mildly reduced. Golgi Staining for Dendritic Spine Density: We used Golgi staining to assess dendritic spine density in the hippocampal region of mice and found that it was only mildly reduced in the circHomer1 interference group. Transmission Electron Microscopy for Axonal Damage: We observed axonal damage in hippocampal neurons using transmission electron microscopy. In POD-like mice induced by surgery, there were no significant differences in axon diameter and myelinated nerve fiber diameter. However, the axon diameter in POD-like mice induced by surgery was significantly impaired and altered, with some layers showing loose structure, and local axon atrophy and narrowing were evident. In the circHomer1 interference group, only significant edema under the axolemma and minor changes were observed.

To explore how circHomer1 interference alleviates postoperative cognitive impairment in elderly mice, we used qRT–PCR to further determine the expression levels of circHomer1 and Homer1 subtypes in the control group, surgery-alone group, surgery + saline injection group, and surgery + circHomer1 interference group. The results showed that the expression level of Homer1a was significantly increased in the surgery-alone and surgery + saline injection groups, while the expression level of Homer1b was significantly decreased in these groups but significantly increased in the surgery + circHomer1 interference group. These results suggest that circHomer1 interference can reduce the degree of cognitive impairment, possibly by interferencing Homer1b. This is consistent with the findings of Hafez et al. (2022), who used the orbitofrontal cortex (OFC) and stem cell-derived neuronal cultures from patients with mental disorders.

To further investigate the role of Homer1b, we designed experiments to both overexpress and silence Homer1b. We observed that in the Homer1b interference group, the expression level of Homer1b was reduced, while in the lentivirus-mediated overexpression group, the expression level of Homer1b increased, as determined by qRT–PCR. Previous studies have shown that Homer1a is an acute-phase product, and its expression typically increases following an emergency response (de Bartolomeis et al., 2022; Miletic et al., 2005), a finding that was also confirmed in our study, It negatively regulates mGluR5 signaling by competitively binding to mGluR5 and disrupting the constitutive Homer1b/c–mGluR5 scaffolding complex. Under postoperative surgical stress, aberrant elevation of Homer1a may interfere with the structural and functional integrity of glutamatergic synapses, impair synaptic plasticity, and ultimately contribute to deficits in learning, memory, and cognitive function. However, Homer1b is constitutively expressed (Tappe and Kuner, 2006), suggesting that Homer1b may be a key molecule in regulating the Homer1/mGluR5 signaling pathway. Therefore, we further explored the effects of Homer1b overexpression and silencing on the mRNA expression of other Homer1 subtypes. Our results showed that compared to the surgery + saline injection group, overexpression of Homer1b had no significant effect on the expression of Homer1a and only a minor effect on Homer1c expression. Similarly, silencing Homer1b did not significantly change the expression levels of Homer1a and Homer1c compared to the surgery + saline injection group. These data indicate that the overexpression and silencing of Homer1b have minimal effects on the expression of other Homer1 subtypes, and the impact on upstream and downstream signaling is primarily due to Homer1b itself rather than synergistic effects with other subtypes. Subsequently, in the fear conditioning experiments, we found that compared to the surgery + NS group, the total percentage of freezing in the contextual test on the third day after surgery was significantly reduced in the Homer1b interference group, while it was significantly increased in the Homer1b overexpression group during the contextual test in the second week after surgery. In the open field test, mice with Homer1b interference showed a significant decrease in the distance traveled in the center area, while mice with Homer1b overexpression showed a significant increase in the distance traveled in the center area. These data suggest that the effects of Homer1b overexpression and silencing are specific; silencing Homer1b exacerbates cognitive impairment in mice, while overexpression of Homer1b protects cognitive function.

Based on previous studies (Qiu et al., 2015), we used a 10-micron Hamilton syringe and a stereotactic apparatus to inject 25 nanomoles (2 microliters) of the mGluR5 agonist CHPG into the vCA1 region of the mGluR5 agonist group in mice. Under the same conditions, the mGluR5 antagonist group received 25 nanomoles (2 microliters) of MPEP (Byrnes et al., 2012; Martínez-Rivera et al., 2013; Phillips et al., 2010; Zhang et al., 2021). Subsequently, we used Western blotting to detect the expression of mGluR5 protein in both groups. Compared to the blank control group, the expression level of mGluR5 protein significantly increased in the agonist group. In contrast, the expression level of mGluR5 protein significantly decreased in the antagonist group. The total percentage of freezing in the contextual test on the third day after surgery significantly increased in the mGluR5 agonist group, while it significantly decreased in the mGluR5 antagonist group. The open field test showed that the distance traveled in the center area by mice in the mGluR5 agonist group was significantly greater than that of mice in the mGluR5 antagonist group. These data indicate that inhibition of mGluR5 receptor expression exacerbates cognitive impairment in mice, while overexpression of mGluR5 receptors also alleviates cognitive impairment, consistent with previous studies (Tronson et al., 2010).

### Limitations

Although this study did not perform dual-luciferase reporter assays and lacks direct evidence for the pathway of circHomer1 inhibiting Homer1b, the study by Hafez et al. (2022) has clearly shown, using RNA antisense purification and other methods, that circHomer1 directly binds to Homer1b mRNA. Lack of LTP Detection: We did not measure long-term potentiation (LTP) in the hippocampus, primarily because the older age of the mice made it difficult to maintain cellular activity, thus failing to obtain satisfactory LTP results. Funding Constraints: Due to funding limitations, we did not perform immunofluorescence or transmission electron microscopy experiments at the positive and negative regulation levels of Homer1b and mGluR5. Therefore, we could not obtain data on changes in hippocampal tissue following the aforementioned drug treatments. Homer1a may also be involved in the pathogenesis of POD-like postoperative neurocognitive impairment, as it significantly increases in the surgery group and the surgery +NS group. It should be noted that while previous studies have convincingly demonstrated that circHomer1 is predominantly expressed in neurons within the hippocampus (Cai et al., 2026), It should be noted that the present study did not examine glial expression of circHomer1 (requiring future FISH with cell-type-specific markers), direct circHomer1–Homer1b interaction, microglial activation, or broader downstream mGluR5 signaling. Consequently, the proposed circHomer1-Homer1b-mGluR5 axis remains partially resolved and needs further investigation using complementary approaches (e.g., RNA pull-down, cell-type-specific knockdown, microglial depletion, phosphoproteomics). We hope to clarify this issue in future work.

## Conclusion

These findings suggest that circHomer1 may contribute to POD-like early postoperative cognitive impairment in aged mice, potentially through modulation of Homer1b/mGluR5-associated signaling in the vCA1 region. However, direct circHomer1-Homer1b interaction were not tested in this study and require further investigation.

## Data Availability

The original contributions presented in this study are included in the article/Supplementary material, further inquiries can be directed to the corresponding author.
