# Supplementary material for: CircHomer1 may contribute to postoperative cognitive impairment by modulating Homer1b/mGluR5-associated signaling in the vCA1 region of aged mice
Source: Front Mol Neurosci. 2026 Jul 6;19:1859800. doi: 10.3389/fnmol.2026.1859800 (PMC13386422; doi:10.3389/fnmol.2026.1859800)
Supplement: Supplementary file 1 [file Table_1.docx]

**Supplementary Table 1. The forward and reverse primers used for qRT‒PCR.**

| mmu-circRNA_26701 | Forward primer | GCATTGCCATTTCCACATAGG |
| --- | --- | --- |
|  | Reverse primer | GTGCTGAAGATAGGTTGCTCCC |
| Homer1a | Forward primer | ATGGGACAAGACGATGAGAGAAC |
|  | Reverse primer | TGCTGAATTGAATGTGTACCTATG |
| Homer1b/c/d | Forward primer | AGTCCACTGCCAATGTGAAGC |
|  | Reverse primer | TTTCAGGGTCTCTTCCAGTTCC |
| Homer1c | Forward primer | GCCCTCTCTCATGCTAGTTCAG |
|  | Reverse primer | GCAGTGAGTTTGGCATTGTTG |
| Homer1d | Forward primer | GTAAAGGCAGCCCAACAACG |
|  | Reverse primer | TCTTTGTGTTCGGGTCAATCTG |
| β-actin | Forward primer | GTGGCCGAGGACTTTGATTG |
