# Supplementary material for: CircHomer1 may contribute to postoperative cognitive impairment by modulating Homer1b/mGluR5-associated signaling in the vCA1 region of aged mice
Source: Front Mol Neurosci. 2026 Jul 6;19:1859800. doi: 10.3389/fnmol.2026.1859800 (PMC13386422; doi:10.3389/fnmol.2026.1859800)
Supplement: Supplementary file 2 [file Table_2.docx]

**Supplementary Table 2. siRNA sequences.**

| si-m-Homer1_001 | GCATTGCCATTTCCACATA |
| --- | --- |
| si-m-Homer1_002 | CACAAAGAAGAACTGGGTA |
| si-m-Homer1_003 | CACCAAACATGACATTTAC |
| si-mmu_circRNA_26701_001 | AAUCUCAGGGAGCAACCUA |
| si-mmu_circRNA_26701_002 | AUCUCAGGGAGCAACCUAU |
| si-mmu_circRNA_26701_003 | AUCUCAGAGGAGCAACCUA |
